# Supplementary material for: MetaRibo-Seq measures translation in microbiomes
Source: Nat Commun. 2020 Jun 29;11:3268. doi: 10.1038/s41467-020-17081-z (PMC7324362; doi:10.1038/s41467-020-17081-z)
Supplement: Supplementary file 10 — Supplementary Data 7 [file 41467_2020_17081_MOESM10_ESM.zip › File2/Confidence_High_Taxonomy/199690_out.krona.html]

Javascript must be enabled to view this page.

members
magnitude
magnitudeUnassigned
count
unassigned
taxon
rank

199690\_out

304

superkingdom
304
2

7
phylum
1239

186801
class
4

186802
4
order

541000
family
4

216851
genus
2

853

SRS052697\_contig\_number\_43852SRS148091\_contig\_number\_1496
2
species

1263
genus
2

species
1

SRS016954\_contig\_number\_8982
2293169

41978

SRS142599\_contig\_number\_29877
1
species

526524
class
3

526525
order
3

3
family
128827

3
genus
135858

100886
3
species

SRS018888\_contig\_number\_1804SRS078665\_contig\_number\_8073SRS097889\_contig\_number\_15924

297
phylum
976

class
297
200643

order
297
171549

family
286
815

286

SRS011084\_contig\_number\_20867SRS014287\_contig\_number\_9538SRS015065\_contig\_number\_contig-100\_854.175856SRS015431\_contig\_number\_30860SRS015579\_contig\_number\_7152SRS015794\_contig\_number\_11131SRS016753\_contig\_number\_4659SRS018049\_contig\_number\_contig-100\_242.243SRS018133\_contig\_number\_contig-100\_2019.81654SRS019445\_contig\_number\_12166SRS020271\_contig\_number\_560SRS021948\_contig\_number\_28706SRS022713\_contig\_number\_6955SRS024009\_contig\_number\_992SRS046502\_contig\_number\_6313SRS047014\_contig\_number\_contig-100\_1062.169488SRS050925\_contig\_number\_contig-100\_832.172823SRS052027\_contig\_number\_contig-100\_1873.113018SRS053398\_contig\_number\_contig-100\_1908.81740SRS055982\_contig\_number\_752SRS056259\_contig\_number\_contig-100\_2847.160355SRS056537\_contig\_number\_63398SRS058770\_contig\_number\_23741SRS063040\_contig\_number\_2630SRS074670\_contig\_number\_1099SRS075878\_contig\_number\_564SRS075963\_contig\_number\_contig-100\_921.921SRS075984\_contig\_number\_contig-100\_942.143784SRS1041140\_contig\_number\_10121SRS1041164\_contig\_number\_2849SRS104311\_contig\_number\_19028SRS143598\_contig\_number\_263SRS143722\_contig\_number\_10942SRS144603\_contig\_number\_5883SRS147766\_contig\_number\_1272SRS148721\_contig\_number\_18763SRS149879\_contig\_number\_18149SRS893358\_contig\_number\_contig-100\_1075.66872
38
genus
816

93974

SRS015264\_contig\_number\_3509
1
species

1

SRS019286\_contig\_number\_5138
species
2292933

1

SRS011239\_contig\_number\_18281
species
310298

2
species

SRS075078\_contig\_number\_2993SRS1041141\_contig\_number\_1906
1739517

species
152

SRS011134\_contig\_number\_16651SRS013158\_contig\_number\_963SRS013638\_contig\_number\_11782SRS013800\_contig\_number\_446SRS013951\_contig\_number\_32319SRS014313\_contig\_number\_contig-100\_914.48130SRS014459\_contig\_number\_17724SRS014855\_contig\_number\_16382SRS014923\_contig\_number\_22371SRS015369\_contig\_number\_contig-100\_89.57693SRS015578\_contig\_number\_22169SRS015694\_contig\_number\_24194SRS015782\_contig\_number\_35594SRS016095\_contig\_number\_13166SRS016840\_contig\_number\_902SRS016897\_contig\_number\_356SRS017103\_contig\_number\_34533SRS017247\_contig\_number\_4820SRS017433\_contig\_number\_3634SRS017521\_contig\_number\_8086SRS017701\_contig\_number\_9185SRS017745\_contig\_number\_1343SRS017821\_contig\_number\_13515SRS018541\_contig\_number\_9422SRS018984\_contig\_number\_5743SRS019030\_contig\_number\_1346SRS019161\_contig\_number\_2919SRS019601\_contig\_number\_25031SRS019808\_contig\_number\_19507SRS020233\_contig\_number\_48794SRS020394\_contig\_number\_1260SRS020622\_contig\_number\_2337SRS021153\_contig\_number\_2585SRS021219\_contig\_number\_3875SRS022071\_contig\_number\_12423SRS022524\_contig\_number\_12093SRS023715\_contig\_number\_23822SRS023914\_contig\_number\_14359SRS023971\_contig\_number\_721SRS024331\_contig\_number\_3110SRS024492\_contig\_number\_2798SRS024663\_contig\_number\_9725SRS042628\_contig\_number\_13563SRS043001\_contig\_number\_1675SRS043411\_contig\_number\_2921SRS045826\_contig\_number\_12920SRS046068\_contig\_number\_240SRS046717\_contig\_number\_6815SRS047433\_contig\_number\_11981SRS048981\_contig\_number\_1670SRS049446\_contig\_number\_14352SRS049773\_contig\_number\_16818SRS049896\_contig\_number\_13543SRS049959\_contig\_number\_36351SRS049995\_contig\_number\_9832SRS050026\_contig\_number\_5022SRS050520\_contig\_number\_222SRS050801\_contig\_number\_3719SRS051882\_contig\_number\_contig-100\_843.130005SRS053087\_contig\_number\_2494SRS053335\_contig\_number\_1533SRS053649\_contig\_number\_20533SRS054905\_contig\_number\_1928SRS054956\_contig\_number\_10583SRS056273\_contig\_number\_3391SRS057478\_contig\_number\_651SRS058723\_contig\_number\_2235SRS062427\_contig\_number\_3785SRS063370\_contig\_number\_2541SRS063489\_contig\_number\_7513SRS063518\_contig\_number\_contig-100\_563.218294SRS063928\_contig\_number\_148SRS064232\_contig\_number\_1125SRS064557\_contig\_number\_1597SRS064645\_contig\_number\_800SRS064757\_contig\_number\_contig-100\_389.142238SRS074586\_contig\_number\_2495SRS075773\_contig\_number\_26811SRS075821\_contig\_number\_16430SRS076804\_contig\_number\_12509SRS077231\_contig\_number\_3786SRS077454\_contig\_number\_contig-100\_140.103496SRS077502\_contig\_number\_5556SRS077641\_contig\_number\_contig-100\_663.121894SRS097958\_contig\_number\_11072SRS098571\_contig\_number\_27926SRS100021\_contig\_number\_1435SRS101433\_contig\_number\_12295SRS1041031\_contig\_number\_10220SRS1041037\_contig\_number\_contig-100\_1021.83270SRS1041039\_contig\_number\_9217SRS1041095\_contig\_number\_553SRS1041112\_contig\_number\_5152SRS1041116\_contig\_number\_8783SRS1041118\_contig\_number\_9982SRS1041129\_contig\_number\_11666SRS1041130\_contig\_number\_1160SRS1041134\_contig\_number\_577SRS1041137\_contig\_number\_23374SRS1041145\_contig\_number\_33786SRS1041147\_contig\_number\_779SRS104327\_contig\_number\_384SRS104400\_contig\_number\_50664SRS104693\_contig\_number\_9930SRS1054691\_contig\_number\_10353SRS1055043\_contig\_number\_4420SRS1055069\_contig\_number\_12018SRS1055095\_contig\_number\_116SRS1055099\_contig\_number\_1266SRS140492\_contig\_number\_10962SRS140513\_contig\_number\_3651SRS142503\_contig\_number\_18199SRS142781\_contig\_number\_8973SRS142890\_contig\_number\_31498SRS142923\_contig\_number\_2007SRS143085\_contig\_number\_861SRS143181\_contig\_number\_5312SRS143466\_contig\_number\_29480SRS143523\_contig\_number\_13597SRS143780\_contig\_number\_1170SRS143895\_contig\_number\_18783SRS144135\_contig\_number\_25160SRS144183\_contig\_number\_31211SRS144297\_contig\_number\_14468SRS144506\_contig\_number\_30755SRS144537\_contig\_number\_10614SRS144714\_contig\_number\_17945SRS147271\_contig\_number\_11716SRS147557\_contig\_number\_28070SRS147614\_contig\_number\_2642SRS147653\_contig\_number\_1562SRS147919\_contig\_number\_14133SRS148253\_contig\_number\_2520SRS148319\_contig\_number\_1665SRS148424\_contig\_number\_9098SRS148784\_contig\_number\_7800SRS148817\_contig\_number\_435SRS148970\_contig\_number\_656SRS149181\_contig\_number\_19994SRS149244\_contig\_number\_3887SRS150029\_contig\_number\_12315SRS892882\_contig\_number\_11393SRS893230\_contig\_number\_5907SRS893231\_contig\_number\_290SRS893252\_contig\_number\_3594SRS893253\_contig\_number\_116SRS893270\_contig\_number\_14341SRS893292\_contig\_number\_4083SRS893300\_contig\_number\_2746SRS893366\_contig\_number\_13364SRS893373\_contig\_number\_6331SRS893380\_contig\_number\_295
820

species
1

SRS024435\_contig\_number\_10454
291644

2

SRS011405\_contig\_number\_664SRS022609\_contig\_number\_21280
species
28111

4
species

SRS048060\_contig\_number\_1447SRS048164\_contig\_number\_27139SRS077392\_contig\_number\_20789SRS143342\_contig\_number\_5025
47678

671267
species
1

SRS101441\_contig\_number\_contig-100\_1487.1487

1

SRS075021\_contig\_number\_1007
species
357276

1
species

SRS014979\_contig\_number\_20856
1263055

9

SRS015854\_contig\_number\_390SRS019496\_contig\_number\_15403SRS019968\_contig\_number\_9540SRS045244\_contig\_number\_661SRS051610\_contig\_number\_8677SRS076976\_contig\_number\_2634SRS1055049\_contig\_number\_4380SRS1055083\_contig\_number\_6156SRS893295\_contig\_number\_10207
species
821

1
species

SRS077194\_contig\_number\_12920
1946083

1263054
1

SRS024625\_contig\_number\_7406
species

species
10

SRS017859\_contig\_number\_2037SRS019267\_contig\_number\_3631SRS054059\_contig\_number\_contig-100\_67.35420SRS055017\_contig\_number\_28583SRS076756\_contig\_number\_10534SRS1041091\_contig\_number\_9499SRS1041138\_contig\_number\_2233SRS893172\_contig\_number\_8234SRS893187\_contig\_number\_11236SRS893256\_contig\_number\_609
93975


SRS013098\_contig\_number\_6282
1
species
338188

626930
1
species

SRS971275\_contig\_number\_31392

1

SRS077552\_contig\_number\_21290
species
1638779

162156
2

SRS077127\_contig\_number\_2789SRS145497\_contig\_number\_935
species

1263045
1

SRS045713\_contig\_number\_2367
species


SRS015890\_contig\_number\_12355SRS021484\_contig\_number\_1053SRS024075\_contig\_number\_2112SRS098655\_contig\_number\_4472
4
species
817


SRS018427\_contig\_number\_837SRS077335\_contig\_number\_263SRS098073\_contig\_number\_12765SRS103987\_contig\_number\_14789SRS104975\_contig\_number\_15379SRS143148\_contig\_number\_24158
6
species
246787

2292944
species
2

SRS050941\_contig\_number\_5396SRS149325\_contig\_number\_10482


SRS050998\_contig\_number\_12821SRS075716\_contig\_number\_3375
2
species
2292931

1
species

SRS018623\_contig\_number\_11779
329854

species
2

SRS016132\_contig\_number\_359SRS077086\_contig\_number\_950
28116

2292950

SRS1041143\_contig\_number\_contig-100\_3662.46667SRS143372\_contig\_number\_10426SRS149075\_contig\_number\_contig-100\_602.115663
3
species

674529
species
2

SRS016056\_contig\_number\_2616SRS144362\_contig\_number\_3345

species
1

SRS104636\_contig\_number\_1475
2292943

1897051
1
species

SRS016018\_contig\_number\_5223

1929999
species
1

SRS018836\_contig\_number\_25642


SRS018351\_contig\_number\_9208SRS019068\_contig\_number\_17882SRS048870\_contig\_number\_2803SRS077294\_contig\_number\_5004SRS147346\_contig\_number\_11423
5
species
818

1
species

SRS050299\_contig\_number\_contig-100\_405.72065
2292938


SRS097920\_contig\_number\_13095
1
species
1946092

46506

SRS012273\_contig\_number\_10629SRS013687\_contig\_number\_13358SRS014412\_contig\_number\_4042SRS016335\_contig\_number\_12606SRS018936\_contig\_number\_6450SRS020328\_contig\_number\_2848SRS022137\_contig\_number\_2872SRS046369\_contig\_number\_1532SRS078176\_contig\_number\_13661SRS098061\_contig\_number\_1393SRS1054716\_contig\_number\_9386SRS142542\_contig\_number\_2115SRS143876\_contig\_number\_4822SRS143991\_contig\_number\_1813SRS147039\_contig\_number\_386SRS893378\_contig\_number\_5670
16
species

371601
1
species

SRS1055067\_contig\_number\_8902

2292928
species
1

SRS057480\_contig\_number\_3226

2292936
4

SRS014683\_contig\_number\_775SRS019397\_contig\_number\_9304SRS101268\_contig\_number\_568SRS147977\_contig\_number\_5032
species

family
3
2005525

genus
375288

SRS015960\_contig\_number\_4761
3
1

1
species

SRS019693\_contig\_number\_2977
387661

46503

SRS050752\_contig\_number\_11121
1
species

171550
family
8

239759
genus
8

1118061

SRS049402\_contig\_number\_2929
1
species


SRS016203\_contig\_number\_1871SRS052078\_contig\_number\_770SRS065504\_contig\_number\_9076
3
species
1263035

1
species

SRS104084\_contig\_number\_5632
1896970

28117
species
2

SRS893279\_contig\_number\_5043SRS893342\_contig\_number\_872

1262693
1

SRS017916\_contig\_number\_10167
species
